# Monthly Variations in Colorectal Cancer Screening Tests Among Federally Qualified Health Center Patients in Missouri: Quality Improvement Project

**DOI:** 10.2196/64809

**Published:** 2025-03-19

**Authors:** Jane A McElroy, Jamie B Smith, Kevin D Everett

**Affiliations:** 1 Family and Community Medicine Department, University of Missouri, 1 Hospital Dr DC032.00, Columbia, MO, 65212, United States, 1 5738824993

**Keywords:** colorectal cancer screening, federally qualified health center, FQHC, fecal immunochemical test, FIT, FIT-DNA, colorectal cancer, CRC, cancer, cancer screening, colonoscopy, United States, health center, quality improvement

## Abstract

**Background:**

Cancer is the second leading cause of death in the United States. Compelling evidence shows screening detects colorectal cancer (CRC) at earlier stages and prevents the development of CRC through the removal of precancerous polyps. The Healthy People 2030 goal for CRC screening is 68.3%, but only 36.5% of Missouri federally qualified health center patients aged 50‐75 years are up-to-date on CRC screening. For average risk patients, there are three commonly used screening tests in the United States—two types of stool tests collected at home (fecal immunochemical test [FIT]–immunochemical fecal occult blood test [FOBT] and FIT-DNA, such as Cologuard) and colonoscopies completed at procedural centers.

**Objective:**

This study aims to examine variation by month for the three types of CRC testing to evaluate consistent patient care by clinical staff.

**Methods:**

Data from 31 federally qualified health center clinics in Missouri from 2011 to 2023 were analyzed. A sample of 34,124 unique eligible “average risk” patients defined as persons not having a personal history of CRC or certain types of polyps, family history of CRC, personal history of inflammatory bowel disease, and personal history of receiving radiation to the abdomen or pelvic to treat a previous cancer or confirmed or suspected hereditary CRC syndrome. Another eligibility criterion is that patients need to be seen at least once at the clinic to be included in the denominator for the screening rate calculation. Descriptive statistics characterize the sample, while bivariate analyses assess differences in screening types by month.

**Results:**

Completion of CRC screening yielded statistically significant differences for patients completing the different types of CRC screening by month. October-January had the highest proportions of patients (644-680 per month, 8.5%‐10.2%) receiving a colonoscopy, while February-April had the lowest (509-578 per month, 6.9%‐7.8%), with 614 being the average monthly number of colonoscopies. For FIT-FOBT, June-August had the higher proportions of patients receiving this test (563-613 per month, 8.9%‐9.6%), whereas December-February had the lowest (453-495 per month, 7.1%‐8%), with 541 being the average monthly number of FIT-FOBT kits used. For FIT-DNA, March was the most popular month with 11.3% (n=261 per month) of patients using the Cologuard test, followed by April, May, and November (207-220 per month, 8.7%‐9.4%), and January and June (168-171 per month, 7.2%-7.3%) had the lowest proportion of patients using Cologuard, with 193 being the average monthly number of FIT-DNA kits used. Combining all tests, February had the fewest CRC tests completed (1153/16,173, 7.1%).

**Conclusions:**

Home-based tests are becoming popular, replacing the gold standard colonoscopy, but need to be repeated more frequently. Monthly variation of screening over the course of a year suggests that CRC screening efforts and patient care may be less than ideal. Months with lower rates of screening for each type of CRC test represent opportunities for improving CRC screening.

## Introduction

Colorectal cancer (CRC) is the third most common cancer in the United States and the second leading cause of cancer deaths [1]. Evidence shows that screening detects CRC at earlier stages, and its development can be prevented by removing precancerous polyps. For average risk patients, there are three common screening tests—two types of stool tests collected at home (fecal immunochemical test [FIT]–immunochemical fecal occult blood test [FOBT] and FIT-DNA, like Cologuard) and colonoscopies completed at procedural centers. The revised Healthy People 2030 goal for CRC screening among people aged 45‐75 years changed from 74.4% to 68.3% [2]. Federally qualified health centers (FQHCs) provide low-cost care for approximately 30 million people, and 90% of FQHCs’ patient population (n=17,562,189) have an income less than 200% of the federal poverty level [3,4]. The CRC screening rate of patients using FQHCs in Missouri (n=95,191) is 36.5% compared to 74.1% for patients not using FQHCs (n=1,657,026) [5].

Colonoscopy is considered the gold standard of CRC screening since precancerous polyps can be removed at the time of the test, preventing cancer. However, numerous patient and health system barriers to colonoscopies have been identified [6]. Home-based testing is becoming more common, and FIT-DNA use has increased post COVID-19 [7]. The increased FIT-DNA use may reflect patient preference for home-based testing that does not incur being wait-listed for months to get a colonoscopy [8]. Additionally, the manufacturer of FIT-DNA provides a full service in facilitating patients’ completion of the test. This service includes a patient follow-up to encourage returning the kits and results sent directly to the patient’s electronic medical record. For the FIT tests, a clinic is responsible for patient follow-ups regarding stool collection and sending the kit in for analysis [9].

Since screening opportunities take place at patients’ routine visits to health centers, determining screening variation by month can assist health care systems adjust outreach efforts, targeting low use months to establish consistently high CRC screening opportunities throughout the year.

### Objective

This quality improvement project aims to determine if there is variation in the 3 types of CRC testing by month. Identifying variations by month can support targeted attention. The global aim of the quality improvement project was to support FQHCs’ in providing CRC screening opportunities with consistent screening rates each month.

## Methods

### Overview

Starting in 2020 as part of a 5-year Centers for Disease Control and Prevention–funded quality improvement program, our project supported eight health care systems’ initiation or enhancement of four evidence-based interventions to increase CRC screening rates of age-eligible patients using a practice facilitator model. As part of this quality improvement program, up to 4 years of annual data on CRC screening by type and date of completed CRC test for the eligible patient population in the selected health care system were available. Patient characteristics including age, race/ethnicity, primary language, and sex were gathered. Screening compliance was defined as a colonoscopy recommended every 10 years, FIT-FOBT every year, and FIT-DNA every 3 years. Screened for CRC was defined as having a medical record of being up-to-date on one of the three types of tests. For this analysis, eligible patients were aged 50‐75 years with no prior diagnosis of CRC, adenomatous polyps, or inflammatory bowel disease, and no personal diagnosis or family history of known genetic disorders that predispose them to a high lifetime risk of CRC such as Lynch syndrome or familial adenomatous polyposis [10]. Descriptive statistics characterize the sample, while bivariate analyses assess differences in screening types by month. While examining monthly CRC screening rates, data were limited to exclude years where fewer than 10 screenings occurred for any given month. Monthly totals were first calculated, and the average number of tests across all months was used to calculate the average percentage change (increase or decrease) month to month. A *χ*^2^ test for equal proportions of the CRC screening tests by month among the 3 types of CRC tests was then examined. Month was chosen as the unit of analysis since it is easily understood, helping plan and implement activities. A weekly analysis has fewer observations leading to less stable numbers, and holidays influence the days in any week. SAS 9.4 (SAS Institute) was used for the analysis.

### Ethical Considerations

This project was approved by University of Missouri’s Institutional Review Board (IRB 2034264), which allowed analysis of clinical data extracted from electronic medical records without additional consent for the secondary analysis. The data were deidentified for the analysis. All data were transmitted and stored in a Health Insurance Portability and Accountability Act (HIPAA)–compliant secure system (REDCap) [11].

## Results

A total of 31 clinics servicing predominately rural residents yielded 34,124 unique eligible patients from 2011 to 2023. Among these, 6238 (18.3%) were up to date on their CRC screening, another 5170 (15.2%) had received a CRC screening at some time in the past but were not up to date, and the remaining 22,716 (66.6%) patients had no record of being screened for CRC. Most participants were 50‐64 years old (n=24,014, 70.4%), were female (n=19,229, 56.4%), used English as their primary language (n=31,686, 92.9%), and were White (n=27,677, 81.1%; Table S1 in Multimedia Appendix 1). Fewer participants younger than 65 years were up to date on their CRC screening than those 65 years and older. Patients with the highest proportion of ever being screened were Hispanic (837/2032, 41.2%), compared to White (9391/27,677, 33.9%) and Black (533/1385, 38.5%), but fewer Hispanic participants (n=260, 12.8%) were up to date compared to White (n=5386, 19.5%) and Black (n=268, 19.4%) participants (Table S1 in Multimedia Appendix 1). The FQHC systems in this analysis served 87% of patients who were at or below 200% of the federal poverty guidelines. Most clinics (n=28, 90.3%) were located in rural areas of Missouri. Among the clinics, the 2023 annual CRC screening rates ranged from 13.7% to 63.1% (62/451 and 238/377 eligible patients, respectively).

**Figure 1.**
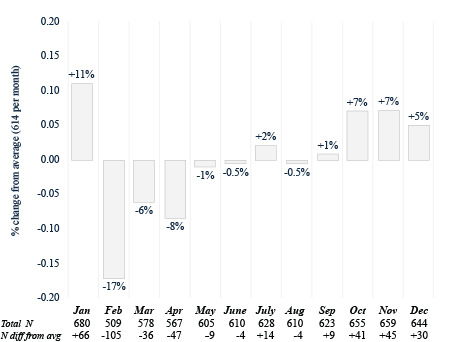
Colonoscopy by month (2014‐2023).

**Figure 2.**
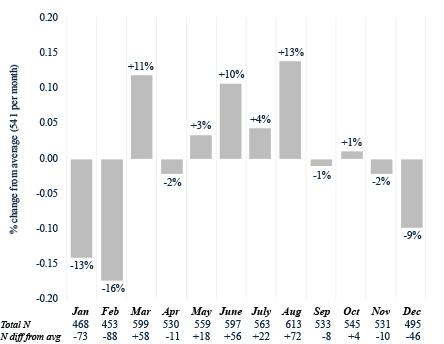
Fecal immunochemistry test–immunochemical fecal occult blood test by month (2017‐2023).

**Figure 3.**
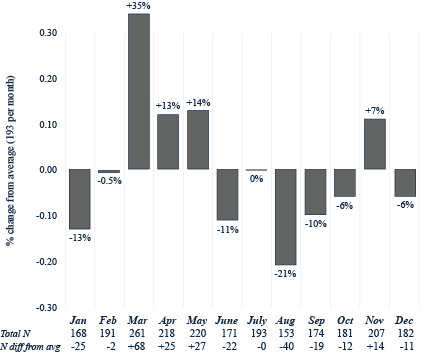
Fecal immunochemistry test–DNA by month (2020‐2023).

Table S2 in Multimedia Appendix 1 breaks down the descriptive statistics on monthly CRC screenings. There were 7368 patients who were up to date on CRC screening by colonoscopy with an average of 614 screenings per month from 2014 to 2023. A *χ*^2^ test for equal proportions found significant differences across monthly colonoscopy screenings (*χ*^2^_11_=38.9; *P*<.001). January was the highest month for colonoscopy screenings (n=680, 11% higher than the average), while February was the lowest (n=509, 17% lower than the average; Figure 1). For FIT-FOBT (n=6486), there were an average of 540.5 screenings per month from 2017 to 2023. A *χ*^2^ test for equal proportions found significant differences across monthly FIT-FOBT screenings (*χ*^2^_11_=51.7; *P*<.001). August was the highest month for FIT-FOBT screenings (n=613, 13% higher than the average) compared to January (n=468, 14% lower than the average) and February (n=453, 16% lower than the average; Figure 2). There were 2319 FIT-DNA screenings, with an average of 193.3 per month from 2020 to 2023. A *χ*^2^ test for equal proportions found significant differences across monthly FIT-FOBT screenings (*χ*^2^_11_=49.2; *P*<.001). March was the highest month (n=261, 35% higher than the average) while January (n=168, 13% lower than the average) and August (n=153, 21% lower than the average) were the lowest months for FIT-DNA testing (Figure 3).

## Discussion

### Principal Findings

Among the 3 types of CRC screening for average risk patients seen at our FQHCs in the United States, no test was completed consistently by month, and each test had different peak months of completion. We were not able to find any research that compared variation by month in CRC screening test types of colonoscopy, FIT-FOBT, and FIT-DNA. To our knowledge, this is the first study that provides results of CRC screening type by month.

As reflected in our screening choices by patients seen at FQHC clinics, home-based CRC screening increased during the COVID-19 pandemic’s closures of specialty care including elective procedures (eg, colonoscopies) [7]. This change in CRC screening options allowed for testing at the discretion of the patient rather than appointment availability.

### Strengths and Limitation

One strength of this study was evaluating patients over 12 years from several FQHCs. These data were snapshots of each year’s CRC screening behavior by the health care systems. This also captured screening behavior before and after the pandemic.

One limitation of this study was our inability to explain the variability by month of the different screening tests. For example, FIT-DNA and FIT-FOBT tests peaked in CRC awareness month in March but not colonoscopies. Additionally, while the results are informative, only a simple analysis of screening variability was performed, which excluded an examination of temporal changes over time.

The preferences of clinicians on which CRC screening test is recommended and their patient care style were not captured. For example, some clinicians only recommend colonoscopy [12-14]; however, some patients who decline a colonoscopy [15] would be willing to complete a home-based CRC screening test if offered. Further reasons for CRC screening refusal of any test were also not captured. These could be a factor in the CRC test variation by month.

### Future Direction

Among the selected participant characteristics, attention is needed on those younger than 65 years to encourage CRC screening. Similarly, while 41.2% of Hispanic participants showed a positive attitude toward CRC screening, only 12.8% were up to date with their screening. This suggests that tailored campaigns and outreach programs could encourage greater participation in CRC screening. For all populations, screening matters since the variance in testing over a year can impact the health care system’s capacity for timely preventive patient care. Gastroenterologist availability to complete colonoscopies may be limited in some regions of the country, but home-based tests can be completed each month [8]. Undoubtedly, individual-level barriers influence CRC screening rates, such as transportation, medical mistrust, financial issues, and low health literacy [16]. However, organizational factors, including monitoring and feedback, have been identified as implementation facilitators [16]. Rockwell and colleagues [6] described health system barriers, especially for colonoscopies, as sludge, “frictions or administrative burdens that make it difficult for people to attain what they want or need.” Providing clinical staff information on completed CRC screening rates by month for each test type may facilitate addressing these “sludge” issues and increase CRC screening [8,17].

## Supplementary material

10.2196/64809 Multimedia Appendix 1 Patient characteristics and descriptive statistics for monthly screenings.

## Abbreviations

CRC: colorectal cancer
FIT: fecal immunochemical test
FOBT: immunochemical fecal occult blood test
FQHC: federally qualified health center
HIPAA: Health Insurance Portability and Accountability Act
