# Supplementary material for: Monthly Variations in Colorectal Cancer Screening Tests Among Federally Qualified Health Center Patients in Missouri: Quality Improvement Project
Source: JMIR Cancer. 2025 Mar 19;11:e64809. doi: 10.2196/64809 (PMC11941909; doi:10.2196/64809)
Supplement: Multimedia Appendix 1 [file cancer-v11-e64809-s001.docx]

**Table S1.** Characteristics of Federal Qualified Health Center Colorectal Cancer Screening Eligible Patients by Screening Status

|  |  | | *Screening Status* | | | | | | | | |
| --- | --- | --- | --- | --- | --- | --- | --- | --- | --- | --- | --- |
|  | *Overall* | | *Up to Date* | | | *Partial Screening* | | | *No Screen Record* | | |
|  | *N* | *%* | *N* | *Row %* | *Col %* | *N* | *Row %* | *Col %* | *N* | *Row %* | *Col %* |
| *Overall* | 34124 | 100.00 | 6238 | 18.28 | 100.00 | 5170 | 15.15 | 100.00 | 22716 | 66.57 | 100.00 |
| Age |  |  |  |  |  |  |  |  |  |  |  |
| 50-54 | 7770 | 22.77 | 1028 | 13.23 | 16.48 | 883 | 11.36 | 17.08 | 5859 | 75.41 | 25.79 |
| 55-59 | 8166 | 23.93 | 1424 | 17.44 | 22.83 | 1206 | 14.77 | 23.33 | 5536 | 67.79 | 24.37 |
| 60-64 | 8078 | 23.67 | 1565 | 19.37 | 25.09 | 1231 | 15.24 | 23.81 | 5282 | 65.39 | 23.25 |
| 65-69 | 5720 | 16.76 | 1221 | 21.35 | 19.57 | 1065 | 18.62 | 20.60 | 3434 | 60.03 | 15.12 |
| 70-75 | 4390 | 12.86 | 1000 | 22.78 | 16.03 | 785 | 17.88 | 15.18 | 2605 | 59.34 | 11.47 |
| *Sex* | 19229 | 56.35 |  |  |  |  |  |  |  |  |  |
| Females |  |  | 3666 | 19.06 | 58.77 | 2965 | 15.42 | 57.35 | 12598 | 65.52 | 55.46 |
| Males | 14886 | 43.62 | 2572 | 17.28 | 41.23 | 2205 | 14.81 | 42.65 | 10109 | 67.91 | 44.50 |
| Unknown | 9 | 0.03 | . | . | . | . | . | . | 9 | 100.00 | 0.04 |
| *Language* | 31686 | 92.86 |  |  |  |  |  |  |  |  |  |
| English |  |  | 5948 | 18.77 | 95.35 | 4529 | 14.29 | 87.60 | 21209 | 66.93 | 93.37 |
| Other | 2438 | 7.14 | 290 | 11.89 | 4.65 | 641 | 26.29 | 12.40 | 1507 | 61.81 | 6.63 |
| Race |  |  |  |  |  |  |  |  |  |  |  |
| White | 27677 | 81.11 | 5386 | 19.46 | 86.34 | 4005 | 14.47 | 77.47 | 18286 | 66.07 | 80.50 |
| Black | 1385 | 4.06 | 268 | 19.35 | 4.30 | 265 | 19.13 | 5.13 | 852 | 61.52 | 3.75 |
| Hispanic | 2032 | 5.95 | 260 | 12.80 | 4.17 | 577 | 28.40 | 11.16 | 1195 | 58.81 | 5.26 |
| Other | 887 | 2.60 | 126 | 14.21 | 2.02 | 170 | 19.17 | 3.29 | 591 | 66.63 | 2.60 |
| Unknown | 2143 | 6.28 | 198 | 9.24 | 3.17 | 153 | 7.14 | 2.96 | 1792 | 83.62 | 7.89 |

**Table S2**. Descriptive statistics for monthly CRC Screenings

| **Colonoscopies** | *Jan* | *Feb* | *Mar* | *April* | *May* | *June* | *July* | *Aug* | *Sept* | *Oct* | *Nov* | *Dec* | *Total* |
| --- | --- | --- | --- | --- | --- | --- | --- | --- | --- | --- | --- | --- | --- |
| # of screenings | 680 | 509 | 578 | 567 | 605 | 610 | 628 | 610 | 623 | 655 | 659 | 644 | 7368 |
| average (M) % | 10.2 | 6.9 | 7.8 | 7.6 | 8.0 | 8.3 | 8.5 | 8.4 | 8.3 | 8.5 | 8.7 | 8.8 | 8.3 |
| median | 9.8 | 6.9 | 7.9 | 8.1 | 8.6 | 8.4 | 8.5 | 7.7 | 8.3 | 8.4 | 8.4 | 8.9 | 8.4 |
| sd | 3.1 | 1.4 | 1.1 | 1.6 | 2.1 | 0.5 | 1.3 | 1.3 | 1.2 | 2.0 | 1.4 | 1.6 | 0.8 |
| min | 5.3 | 5.2 | 5.9 | 4.1 | 4.5 | 7.3 | 7.1 | 7.2 | 5.9 | 4.9 | 6.3 | 6.8 | 4.1 |
| max | 16.9 | 9.5 | 9.4 | 9.2 | 10.5 | 9.0 | 11.3 | 10.6 | 10.3 | 11.2 | 10.6 | 11.7 | 16.9 |
| range | 11.6 | 4.3 | 3.4 | 5.1 | 6.0 | 1.7 | 4.2 | 3.4 | 4.4 | 6.3 | 4.3 | 4.9 | 12.7 |
| lowest yr | 2022 | 2022 | 2019 | 2020 | 2020 | 2018 | 2014 | 2015 | 2017 | 2015 | 2014 | 2016 |  |
| highest yr | 2014 | 2020 | 2023 | 2016 | 2023 | 2017 | 2016 | 2014 | 2022 | 2020 | 2021 | 2018 |  |
|  |  |  |  |  |  |  |  |  |  |  |  |  |  |
| **FIT-FOBT** | *Jan* | *Feb* | *Mar* | *April* | *May* | *June* | *July* | *Aug* | *Sept* | *Oct* | *Nov* | *Dec* | *Total* |
| # of screenings | 468 | 453 | 599 | 530 | 559 | 597 | 563 | 613 | 533 | 545 | 531 | 495 | 6486 |
| average (M) % | 7.1 | 7.2 | 8.4 | 7.8 | 8.2 | 9.3 | 8.9 | 9.6 | 8.0 | 8.8 | 8.6 | 8.0 | 8.3 |
| median | 7.7 | 7.9 | 8.9 | 8.0 | 8.9 | 9.8 | 8.9 | 9.4 | 7.9 | 8.3 | 8.7 | 8.0 | 8.3 |
| sd | 2.1 | 2.2 | 2.9 | 2.3 | 2.0 | 1.4 | 1.4 | 2.0 | 1.7 | 2.0 | 1.7 | 2.3 | 0.8 |
| min | 4.4 | 3.8 | 3.5 | 3.6 | 4.5 | 7.9 | 6.5 | 7.3 | 5.2 | 6.5 | 6.6 | 5.9 | 3.5 |
| max | 10.2 | 10.9 | 12.6 | 10.4 | 10.4 | 11.5 | 10.5 | 11.8 | 10.3 | 12.2 | 11.2 | 12.5 | 12.6 |
| range | 5.8 | 7.1 | 9.1 | 6.8 | 5.9 | 3.7 | 4.0 | 4.6 | 5.0 | 5.8 | 4.6 | 6.7 | 9.1 |
| lowest yr | 2018 | 2022 | 2017 | 2020 | 2020 | 2018 | 2023 | 2021 | 2017 | 2023 | 2023 | 2023 |  |
| highest yr | 2020 | 2023 | 2023 | 2021 | 2023 | 2017 | 2017 | 2018 | 2020 | 2017 | 2017 | 2018 |  |
|  |  |  |  |  |  |  |  |  |  |  |  |  |  |
| **FIT-DNA** | *Jan* | *Feb* | *Mar* | *April* | *May* | *June* | *July* | *Aug* | *Sept* | *Oct* | *Nov* | *Dec* | *Total* |
| # of screenings | 168 | 191 | 261 | 218 | 220 | 171 | 193 | 153 | 174 | 181 | 207 | 182 | 2319 |
| average (M) % | 7.3 | 8.2 | 11.3 | 8.9 | 8.7 | 7.2 | 8.8 | 6.4 | 7.7 | 7.8 | 9.4 | 8.3 | 8.3 |
| median | 7.7 | 8.0 | 11.3 | 9.0 | 8.7 | 7.0 | 9.0 | 6.6 | 7.5 | 7.8 | 10.3 | 9.0 | 8.2 |
| sd | 2.5 | 1.9 | 1.0 | 2.6 | 2.3 | 0.9 | 1.9 | 1.6 | 1.4 | 0.9 | 2.7 | 1.9 | 1.3 |
| min | 3.9 | 6.5 | 10.2 | 6.0 | 6.0 | 6.4 | 6.7 | 4.2 | 6.3 | 6.8 | 5.5 | 5.5 | 3.9 |
| max | 9.8 | 10.1 | 12.4 | 11.5 | 11.6 | 8.4 | 10.6 | 8.0 | 9.5 | 9.0 | 11.5 | 9.7 | 12.4 |
| range | 5.9 | 3.7 | 2.2 | 5.6 | 5.7 | 2.0 | 3.9 | 3.8 | 3.2 | 2.2 | 5.9 | 4.1 | 8.5 |
| lowest yr | 2021 | 2022 | 2021 | 2020 | 2020 | 2022 | 2022 | 2020 | 2021 | 2023 | 2023 | 2023 |  |
| highest yr | 2020 | 2023 | 2022 | 2023 | 2023 | 2023 | 2021 | 2021 | 2020 | 2022 | 2021 | 2022 |  |
|  |  |  |  |  |  |  |  |  |  |  |  |  |  |

FIT-FOBT (fecal immunochemistry test/immunochemical fecal occult blood test); FIT-DNA (fecal mmunochemistry test-DNA)

Range of years: Colonoscopies (2014-2023), FIT-FOBT (2017-2023), FIT-DNA (2020-2023)
